# Exploring the practice of nutritional support during hospitalization across physicians, dietitians, and pharmacists based in Saudi Arabia

**DOI:** 10.3389/fnut.2023.1149727

**Published:** 2023-05-24

**Authors:** Sarah M. Ajabnoor, Sara Zaher, Rania Malatani, Hani Jawa

**Affiliations:** ^1^Clinical Nutrition Department, Faculty of Applied Medical Sciences, King Abdulaziz University, Jeddah, Saudi Arabia; ^2^Clinical Nutrition Department, Faculty of Applied Medical Sciences, Taibah University, Madinah, Saudi Arabia; ^3^Department of Pharmacy Practice, Faculty of Pharmacy, King Abdulaziz University, Jeddah, Saudi Arabia; ^4^Department of Medicine, King Abdulaziz University, Jeddah, Saudi Arabia

**Keywords:** nutritional support, enteral nutrition, parenteral nutrition, nutritional support team, practice

## Abstract

**Background:**

Nutritional support has a pivotal role in preventing and treating malnutrition. Recognizing the gaps in nutritional support practice can aid the development of tailored nutritional protocols. Therefore, this study aimed to assess the current practices, attitudes, and perceptions related to nutritional support for hospitalized patients in one of the largest Middle Eastern countries.

**Methods:**

A cross-sectional study was conducted among different healthcare professionals currently working in hospitals in Saudi Arabia and involved in nutritional support practice. Data were collected using convenient sample via a self-administered web-based questionnaire.

**Results:**

A total of 114 participants were included in this study. The majority were dietitians (54%), followed by physicians (33%) and pharmacists (12%), and were from the western region (71.9%). Various attitudes in many practices were observed among the participants. Only 44.7% of the participants had a formal nutritional support team. The mean confidence level of all respondents was significantly higher for enteral nutrition practice (7.7 ± 2.3) than for parenteral nutrition practice (6.1 ± 2.5) (*p* < 0.01). The confidence level for enteral nutrition practice was significantly influenced by nutritional qualification (β = 0.202, *p* < 0.05), type of healthcare facility (β = 0.210, p < 0.05), profession (β = -0.308, *p* < 0.01), and years of experience (β = 0.220, *p* < 0.05).

**Conclusion:**

This study comprehensively assessed various aspects of nutritional support practice in Saudi Arabia. Healthcare practice of nutritional support should be guided by evidence-based guidelines. Professional qualification and training in nutritional support are essential for promoting practice in hospitals.

## Introduction

More than 40% of hospitalized patients are considered malnourished (1). In Saudi Arabia, robust national data on the malnutrition rate in hospitalized patients are lacking. An earlier single-center study that utilized anthropometric data reported malnutrition in up to 34% of hospitalized patients (2). Other studies used the Mini Nutritional Assessment (MNA) tool for reporting malnutrition in hospitalized elderly patients; up to 36.5% of patients were malnourished, while up to 57.8% were at risk for malnutrition (3–5). The malnutrition rate in hospitalized elderly patients was associated with a higher mortality rate and prolonged hospital stay (5). Nevertheless, the prevalence of malnutrition on admission is consistently high (between 40 and 60%) (6). Malnutrition should be identified early during hospital admission, and nutritional care plans should be appropriately initiated. Recently, the American Society for Parenteral and Enteral Nutrition (ASPEN) issued updated evidence-based standards for various nutritional support practices in hospitalized adult patients, which help clinicians deliver safe and efficient nutritional care plans (7). The availability of such guidelines should prevent inappropriate practices of nutritional support, such as late feed initiation, inappropriate parenteral nutrition (PN) prescription for patients who can tolerate enteral nutrition (EN), unmet patient caloric requirements, or poor monitoring of EN/PN-related complications. However, inappropriate practices resulting from insufficient knowledge and nutritional training and poor compliance with available guidelines are still being reported among healthcare professionals (8, 9). In Saudi Arabia, there is a lack of consensus and national guidelines for clinical nutrition practice. Thus, nutritional support protocols for hospitalized patients are warranted.

The efficiency and safety of nutritional support delivery can be optimized with multidisciplinary approaches. Nutritional support teams (NSTs) have been established since 1980 by many hospitals to provide optimal nutrition care for patients receiving EN or PN (10). NSTs usually comprise dietitians, pharmacists, nurses, and physicians. Most assigned leaders for NSTs are either physicians or dietitians (11). All clinicians included in NSTs should gain expertise and undergo training in nutritional support. The recent ASPEN consensus for appropriate PN practices supports the utilization of NSTs comprising healthcare professionals with the expertise to provide proper PN management (12). NST implementation in hospitals is associated with many positive outcomes, including fewer EN/PN-related complications, improved patient safety, and reduced hospital costs (12). NST implementation is also associated with reduced electrolyte abnormalities (i.e., refeeding syndrome) and mortalities in patients receiving PN (13). However, there is a lack of data on NST implementation in hospitals and its outcomes in Saudi Arabia. Another important function of NST implementation is monitoring of patients receiving long-term home nutritional support, which can be complex and challenging (11).

The current nutritional support practices across hospitals in Saudi Arabia are not fully described. There are no national guidelines supporting appropriate nutritional support practice in hospitalized patients. Therefore, this study primarily aimed to assess the current practices, attitudes, and perceptions related to nutritional support for hospitalized patients among physicians, dietitians, and pharmacists in Saudi Arabia.

## Materials and methods

### Study design

This cross-sectional study used an online survey conducted from August 2020 to February 2021 among healthcare professionals working in hospitals in Saudi Arabia.

Registered dietitians and clinical pharmacists currently practicing and physicians routinely involved in nutritional support (i.e., gastroenterologists, surgeons, and critical care intensivists) were included. Other healthcare professionals were excluded.

Data collection was done using convenience sampling technique. The questionnaire was primarily distributed via several platforms, such as the Saudi Gastroenterology Association, the Saudi General Surgery Society, the Saudi Critical Care Society, the Saudi Society for Clinical Nutrition, the Saudi Society of Clinical Pharmacy, and social media. Members of national associations were invited to participate in the study via email with a link for the questionnaire. Because recruitment was online and open, the response rate was not calculated.

### Survey development

The questionnaire comprised six main sections covering 45 items. Included questions were developed by the research team after reviewing previous surveys that investigated different areas of nutritional support practice among physicians and other healthcare professionals working in Canada, the United States, and Europe (14–19). The main sections of the questionnaire were designed to assess the respondent demographic data, structure and performance of the NST, nutritional screening and assessment practices, use of established nutritional support guidelines, attitudes related to initiation and monitoring of nutritional support, and perceptions related to the current knowledge of nutritional support. The questionnaire was electronically created using Google Forms.

For questionnaire validation, pilot testing of the survey was initially conducted among an expert panel, which included one dietitian, two Ph.D holders in clinical nutrition, and two pharmacists. The panel assessed the appropriateness of the questionnaire and the time spent for completing the survey. All feedback was considered by the research investigators. The clinicians who participated in the pilot testing were excluded from the main study survey.

### Ethical approval

The study was approved by the Unit of the Biomedical Ethics Research Committee at King Abdulaziz University in Jeddah, Saudi Arabia (HA-02-J-008). All participants provided an electronic informed consent prior to answering the questionnaire. Statements regarding confidentiality and anonymity were included on the first page of the questionnaire.

### Statistical analysis

Data were downloaded and analyzed using the Statistical Package for the Social Sciences version 23 (SPSS Inc.). The Shapiro–Wilk test was used to assess the normality of continuous variables. Data were presented as either means ± standard deviations or frequencies and percentages.

The Mann–Whitney U-test was used to compare the confidence score between two variables while the Kruskal–Wallis test was used to compare the confidence score between more than two variables.

A stepwise linear regression analysis was performed to identify the factors influencing the healthcare providers’ confidence in practicing nutritional support. The confidence level was used as the outcome variable in the regression models. The dependent variables used in the models were nutritional qualification (yes coded as 1 and no as 0), type of healthcare facility (Ministry of Health hospital coded as 1, military hospital as 2, university teaching hospital as 3, specialized hospital as 4, national guard hospital as 5, medical city as 6, Security Forces Hospital as 7, and private hospital as 8), profession (dietitians coded as 1, pharmacists as 2, and physicians as 3), years of experience (0 years [newly graduated] coded as 1, 2–5 years as 2, 6–10 years as 3, and > 10 years as 4), region (western coded as 1, eastern as 2, central as 3, southern as 4, and northern as 5), and capacity of healthcare facility (<100 beds coded as 1, 100–250 beds as 2, 251–500 beds as 3, and >500 beds as 4).

All performed tests were two-tailed, with a significance level of 95%.

## Results

### Participant characteristics

A total of 140 respondents agreed to participate in the study; of them, only 117 answered yes when asked whether they were involved in nutritional support for hospitalized patients. Nutritional support was defined in the survey as a part of medical therapy that helps in treating and preventing malnutrition and includes EN and PN. Three responses were excluded owing to duplication and incompletion. The final analysis included 114 participants. Of them, 38 (33%) were physicians; 62 (54%), dietitians; and 14 (12%), pharmacists. The majority (71.9%) were from the western region of Saudi Arabia. The participant demographics are described in Table 1.

**TABLE 1:** Demographics of the study participants.

Demographic characteristics		Physicians (*n* = 38)	Dietitians (*n* = 62)	Pharmacists (*n* = 14)	Total (*n* = 114)
		***n* (%)**			
Region	Western region	22 (57.9%)	48 (77.4%)	12 (85.7%)	82 (71.9%)
	Eastern region	3 (7.9%)	5 (8.1%)	2 (14.3%)	10 (8.8%)
	Central region	13 (34.2%)	6 (9.7%)	0 (0.0%)	19 (16.6%)
	Southern region	0 (0.0%)	3 (4.8%)	0 (0.0%)	3 (2.6%)
	Northern region	0 (0.0%)	0 (0.0%)	0 (0.0%)	0 (0.0%)
Years of experience	Newly graduated	1 (2.6%)	3 (4.8%)	0 (50.0%)	4 (3.5%)
	2–5 years	8 (21.1%)	28 (45.2%)	7 (50.0%)	43 (37.7%)
	6–10 years	4 (10.5%)	20 (32.3%)	5 (35.7%)	29 (25.4%)
	More than 10 years	25 (65.8%)	11 (17.7%)	2 (14.3%)	38 (33.3%)
Type of health care facility	Medical cities (e.g., Prince Sultan Medical City)	1 (2.6%)	5 (8.1%)	0 (0.0%)	6 (5.3%)
	Military hospitals	0 (0.0%)	8 (12.9%)	4 (28.6%)	12 (10.5%)
	Ministry of Health (MOH) hospitals	7 (18.4%)	24 (38.7%)	0 (0.0%)	31 (27.2%)
	National guard hospitals	3 (7.9%)	3 (4.8%)	1 (7.1%)	7 (6.1%)
	Private hospitals	10 (26.3%)	8 (12.9%)	0 (0.0%)	18 (15.8%)
	Security Forces Hospital	2 (5.3%)	0 (0.0%)	0 (0.0%)	2 (1.8%)
	Specialized hospitals (King Faisal Specialist Hospital and Research Centre)	6 (15.8%)	6 (9.7%)	3 (21.4%)	15 (13.2%)
	University teaching hospitals	9 (23.7%)	8 (12.9%)	6 (42.9%)	23 (20.2%)
Capacity of health care facility	<100 beds	4 (10.5%)	5 (8.1%)	2 (14.3%)	11 (9.6%)
	100–250 beds	8 (21.1%)	8 (12.9%)	1 (7.1%)	17 (14.9%)
	251–500 beds	8 (21.1%)	29 (46.8%)	4 (28.6%)	41 (36.0%)
	>500 beds	16 (42.1%)	13 (21.0%)	7 (50.0%)	36 (31.6%)
	Don’t know	2 (5.3%)	7 (11.3%)	0 (0.0%)	9 (7.9%)
Nutritional support qualification	ASPEN nutrition support certification	0 (0.0%)	5 (8.1%)	0 (0.0%)	5 (4.4%)
	ESPEN diploma in clinical nutrition and metabolism	0 (0.0%)	3 (4.8%)	0 (0.0%)	3 (2.6%)
	Fellowship in clinical nutrition	2 (5.3%)	6 (9.7%)	1 (7.1%)	9 (7.9%)
	Master of clinical nutrition with focus on nutrition support	0 (0.0%)	10 (16.1%)	0 (0.0%)	10 (8.8%)
	More than one certificate	1 (2.6%)	0 (0.0%)	0 (0.0%)	1 (0.9%)
	Nutrition support training in their local hospital	1 (2.6%)	0 (0.0%)	3 (21.4%)	4 (3.5%)
	Others	1 (2.6%)	3 (4.8%)	0 (0.0%)	4 (3.5%)
	None	33 (86.8%)	35 (56.5%)	10 (71.4%)	78 (68.4%)

Data are presented as numbers and percentages.

### Roles in nutritional support

The participants were asked to report which nutritional support-related tasks they were involved in. All clinicians were relatively involved in determining patient needs for nutritional support and selecting the appropriate feeding route. All dietitians (100%), 85.7% (*n* = 12) of the pharmacists, and only 39.5% (*n* = 15) of the physicians indicated that they participated in estimating the patients’ nutritional requirements.

Approximately 80.6% (*n* = 50) of the dietitians, 60.5% (*n* = 23) of the physicians, and 14.2% (*n* = 2) of the pharmacists reported that they were involved in writing EN orders; 22.5% (*n* = 14), 39.5% (*n* = 15), and 85.7% (*n* = 12) reported that they were involved in writing PN orders, respectively.

The majority of the physicians (65.7%, *n* = 22) and only 12.9% (*n* = 8) of the dietitians indicated that they were involved in the insertion and administration of EN feeding tubes. Only 13.1% (*n* = 5) of the physicians indicated that they participated in placing PN access devices.

**FIGURE 1:**
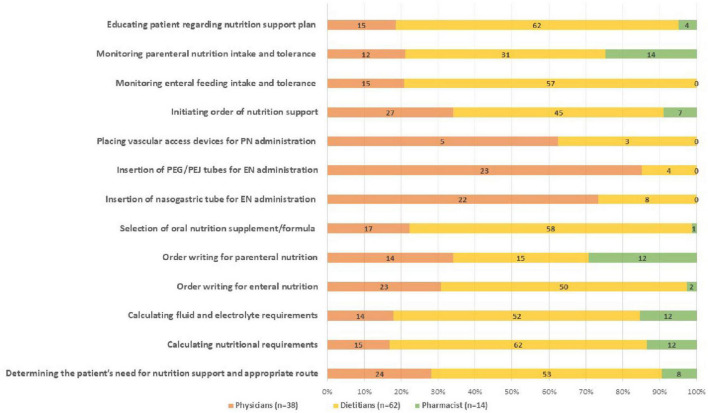
Nutrition support related activities performed by health care providers. This figure shows the frequencies of the nutrition support tasks performed by each profession. Numbers represents the number of participants.

Most dietitians (91.9%, *n* = 62) and pharmacists (100%) indicated their involvement in monitoring EN and PN, respectively. However, a smaller proportion of physicians reported their involvement in monitoring. Figure 1 and Supplementary Table 1 show the main current roles of the respondents in nutritional support.

### Perceptions related to the use of established nutritional support guidelines

Around 78.1% (*n* = 89) of the respondents indicated that they were familiar with the published international guidelines by the ASPEN and/or European Society for Clinical Nutrition and Metabolism (ESPEN). The dietitians and pharmacists expressed greater familiarity with these guidelines.

Approximately 62.3% (*n* = 71) of the participants indicated that written policies for nutritional support provision were available in their institutions. Of them, 45 (63.4%) were involved in writing and updating these policies. The dietitians were highly involved (73%) in updating such policies, while the pharmacists (54.5%) and physicians (33.3%) were relatively less involved. The participants involved in writing policies and protocols for nutritional support reported that the most frequently used guidelines as reference were the ASPEN (47.4%) and ESPEN (38.6%) guidelines. Table 2 shows the physicians’, dietitians’, and pharmacists’ perceptions toward the use of established guidelines.

**TABLE 2:** Perception of health care providers regarding the use of established nutrition support guidelines.

Questions	Answers	Physicians (*n* = 38)	Dietitians (*n* = 62)	Pharmacists (*n* = 14)	Total (*n* = 114)
		***n* (%)**			
(1) Familiar with ASPEN and/or ESPEN guidelines	Familiar	18 (47.3%)	59 (95.1%)	12 (85.7%)	89 (78.1%)
	Not familiar	20 (52.6%)	3 (4.8%)	2 (14.2%)	25 (21.9%)
(2) Availability of written policies and procedures for the provision of nutrition support in my facility (i.e., timing and route of feed initiation, formula selection, assessing patient’s nutrient requirements, and assessing feeding intolerance)*	Available	12 (31.5%)	48 (77.4%)	11 (78.5)	71 (62.3%)
	Not available	10 (26.3%)	8 (12.90%)	0 (0.0%)	18 (15.8%)
	Don’t know	16 (42.1%)	6 (9.67%)	3 (21.4%)	25 (21.9%)
		**Physicians (n = 12)**	**Dietitians (n = 48)**	**Pharmacists (n = 11)**	**Total** **(n = 71)**
		**n (%)**			
(3) Involvement in writing and updating the hospital’s nutrition support policies	Involved	4 (33.3%)	35 (73%)	6 (54.5%)	45 (63.4%)
	Not involved	8 (66.7%)	13 (27%)	5 (45.5%)	26 (36.6%)
(4) Type of guidelines used as a reference in my institution**	ASPEN	2 (5.3%)	43 (68.3%)	9 (64.3%)	54 (47.4%)
	ESPEN	2 (5.3%)	40 (63.5%)	2 (14.3%)	44 (38.6%)
	Other	8 (21.1%)	3 (4.8%)	2 (14.3%)	13 (11.4%)
	Don’t know	0 (0.0%)	2 (3.2%)	0 (0.0%)	2 (1.8%)

Data are presented as numbers and percentages.

*Only participants who answered Yes (*n* = 71) to this question were allowed to proceed to the following questions: participants who answered No or I don’t know were exempted from questions 3 and 4.

**Percentages for each column don’t add to 100 because participants were allowed to choose more than one option. ASPEN, American Society for Parenteral and Enteral Nutrition; ESPEN, European Society of Clinical Nutrition and Metabolism.

### Nutritional screening practices

Approximately 35.96% of the participants were not aware of the screening tool that was routinely used in their hospitals. The Nutrition Risk Screening (NRS 2002) and Malnutrition Universal Screening Tool (MUST) were used routinely by 28.07 and 22.8% of the respondents. Other screening tools, including the MNA, Short Nutritional Assessment Questionnaire (SNAQ), Malnutrition Screening Tool (MST), and Subjective Global Assessment (SGA), were used routinely by fewer respondents.

Only 55 (48.2%) participants reported that screening for malnutrition was routinely conducted in their institutions. Of them, 45.4% selected dietitians as the key persons primarily responsible for the initial screening; 38.2%, nurses; and 16.4%, physicians. The majority (76.4%) reported that screening was routinely initiated on admission with periodic re-screening. Supplementary Table 2 illustrates all participants’ nutritional screening and assessment practices.

### Nutritional assessment practices

The nutritional assessment practices varied among the respondents. Regarding referral to dietitians, 42.1% answered that it was ordered by physicians and 17.5% by nurses after the initial screening. However, 30.7% reported that dietitians assessed all newly admitted patients.

The majority of the respondents (58.8%) relied on anthropometric data (i.e., weight and height) as clinical indicators of nutritional status, while only 28.9% used albumin levels. Few (7.9%) used pre-albumin levels. A small proportion (16.7%) reported that they were not involved in calculating caloric needs. Conversely, 62.3% reported their use of simple weight-based equations, and 18.4% used predictive equations. Only 2.6% indicated that they had access to indirect calorimetry. Supplementary Table 3 shows the nutritional assessment practices by each profession.

### Initiation, monitoring, and documentation of nutritional support plans

#### Initiation practices

Approximately 28.9 and 12.3% of the participants waited 1 and 2 days, respectively, before starting nutritional support for critically ill and hemodynamically stable patients with nil per oral status. Meanwhile, 39.5% waited 3 days. Nearly half (43.9%) of the participants waited 3–5 days before starting PN for well-nourished and stable patients with minimal oral intake or EN (<50% of requirements). Few waited 7–14 days.

#### Monitoring practices

The most common practices for patients on gastric feeding who were complaining of nausea and vomiting were slowing tube feeding (70.2%) and checking the gastric residual volume (GRV) (63.2%). Stopping tube feeding was indicated by 21.9% of the participants. Other practices were also reported by the participants. Nevertheless, about half (51.8%) reported that they routinely measured the GRV to assess EN intolerance.

#### Documentation practices

Only 15.8% indicated that nutritional data were documented manually in their institutions. Meanwhile, data related to nutritional assessment and care plan were documented electronically by 64% of the respondents. Prescription orders for EN and PN were electronically documented by 59.6% and 53.5%, respectively. Table 3 illustrates the main nutritional support practices in Saudi hospitals by each profession.

**TABLE 3:** Nutrition support initiation, monitoring and hospital documentation practices in Saudi hospitals as reported by health care providers.

Questions	Answers	Physicians (*n* = 38)	Dietitians (*n* = 62)	Pharmacists (*n* = 14)	Total (*n* = 114)
		***n* (%)**			
(1) In your practice, in a critically ill and hemodynamically stable patient, after how many days of nil per oral (NPO) status, would you wait before the use of artificial nutrition support?	*1 Day*	8 (21.1%)	22 (35.5%)	3 (21.4%)	33 (28.9%)
	*2 Days*	5 (13.2%)	8 (12.9%)	1 (7.1%)	14 (12.3%)
	*3 Days*	22 (57.9%)	17 (27.4%)	6 (42.9%)	45 (39.5%)
	*Not applicable in my practice*	1 (2.6%)	11 (17.7%)	4 (28.6%)	16 (14.0%)
	*Other*	2 (5.3%)	4 (6.5%)	0 (0%)	6 (5.3%)
(2) In your practice, after how many days of minimal oral intake or enteral nutrition (less than 50% of estimated caloric requirements) by a well-nourished, stable patient, would you initiate parenteral nutrition?	*3-5 Days*	12 (31.5%)	23 (37.0%)	2 (14.2%)	37 (32.4%)
	*7 Days*	20 (52.6%)	22 (35.4%)	7 (50.0%)	49 (42.9%)
	*14 Days*	1 (2.6%)	9 (14.5%)	0 (0.0%)	10 (8.7%)
	*I don’t know*	1 (2.6%)	3 (4.83%)	3 (21.4%)	7 (6.1%)
	*Not applicable in my practice*	4 (10.5%)	5 (8.1%)	2 (14.3%)	11 (9.6%)
(3) In your practice, what would you do if a patient experiences a few nauseas or vomiting with gastric tube feeding?*	*Stop tube feeding*	6 (15.8%)	18 (29.0%)	1 (7.1%)	25 (21.9%)
	*Slow tube feeding*	30 (78.9%)	46 (74.2%)	4 (7.1%)	80 (70.2%)
	*Give promotility agent*	21 (55.3%)	31 (50.0%)	4 (7.1%)	56 (49.1%)
	*Check gastric residual volume*	24 (63.2%)	44 (71.0%)	4 (7.1%)	72 (63.2%)
	*Check tube placement*	28 (73.7%)	28 (45.2%)	2 (7.1%)	58 (50.9%)
	*Place tube to suction*	3 (7.9%)	10 (16.1%)	0 (7.1%)	13 (11.4%)
	*Advance tube*	4 (10.5%)	2 (3.2%)	0 (7.1%)	6 (5.3%)
	*Switch to parenteral feeding*	2 (5.3%)	3 (4.8%)	1 (7.1%)	6 (5.3%)
	*Consult a specialist*	6 (15.8%)	21 (33.9%)	1 (7.1%)	28 (24.6%)
	*Perform a physical examination*	19 (50.0%)	1 (1.6%)	1 (7.1%)	21 (18.4%)
	*Elevate the head of the bed*	18 (47.4%)	48 (77.4%)	0 (7.1%)	66 (57.9%)
	*Check gastric emptying study*	8 (21.1%)	21 (33.9%)	1 (7.1%)	30 (26.3%)
	*Give IV fluids*	12 (31.6%)	5 (8.1%)	4 (7.1%)	21 (18.4%)
	*Other*	2 (5.3%)	5 (8.1%)	0 (7.1%)	7 (6.1%)
	*I Don’t know*	0 (0.0%)	1 (1.6%)	9 (7.1%)	10 (8.8%)
(4) In your practice, do you routinely measure gastric residual volume (GRV) in patients receiving enteral nutrition as a measure of enteral feeding intolerance?	*Yes*	19 (50.0%)	34 (54.8%)	6 (42.9%)	59 (51.8%)
	*No*	10 (26.3%)	26 (41.9%)	0 (0.00%)	36 (31.6%)
	*I Don’t know*	9 (23.7%)	2 (3.2%)	8 (57.1%)	19 (16.7%)
(5) At your institution, which of the following nutritional data is documented or carried out using the hospital’s electronic health record system?*	*Nutrition screening data*	14 (36.8%)	40 (64.5%)	6 (42.9%)	60 (52.6%)
	*Nutrition assessment data*	23 (60.5%)	45 (72.6%)	5 (35.7%)	73 (64.0%)
	*Nutrition care plan*	19 (50.0%)	49 (79.0%)	5 (35.7%)	73 (64.0%)
	*Enteral nutrition order entry*	19 (50.0%)	45 (72.6%)	4 (28.6%)	68 (59.6%)
	*Parenteral nutrition order entry*	21 (55.3%)	30 (48.4%)	10 (71.4%)	61 (53.5%)
	*Nutrition monitoring and evaluation data*	15 (39.5%)	39 (62.9%)	7 50.0%)	61 (53.5%)
	*None of the above (all are done manually)*	8 (21.1%)	8 (12.9%)	2 (14.3%)	18 (15.8%)

Data are presented as numbers and percentages.

*Participants were allowed to choose more than one option.

### Availability of NSTs

The majority of the participants (93.9%) agreed that NSTs were important to the accuracy and efficacy of EN/PN prescription. The next question in the survey aimed to identify the proportion of participants working in hospitals with established NSTs. Approximately 44.7% (*n* = 51) of the clinicians had a formal NST, while 31.5% had none-formal NST (nutritional management was aided by regular communication between disciplines). Fewer participants either had no NST (13.2%) or were not aware (10.5%).

The 51 clinicians who had a formal NST indicated the following as members of the team: dietitians (100%), physicians (96.1%), pharmacists (84.3%), and nurses (74.5%). Conversely, the frequency of NST meetings greatly varied. Approximately 72.5% indicated that NSTs reviewed and reported service performance data. The most common barriers for developing a dedicated NST were a lack of physicians with interest (100%), qualified pharmacist (92.2%), and incentives by hospital administrations (96.1%). Meanwhile, over half (66.7%) indicated no barriers. Table 4 provides an overview of the current practices for NSTs in Saudi hospitals.

**TABLE 4:** Nutrition support team in Saudi hospitals.

Questions	Answers	Total (*n* = 114)
(1) In your opinion, how important is having nutrition support team to the accuracy and efficacy of nutritional prescription for hospitalized patients?	*Very important*	107 (93.9%)
	*Somewhat important*	7 (6.1%)
	*Not important*	0 (0.0%)
(2) Does your hospital have an established multidisciplinary nutrition support team that is currently active?*	*Yes*	51 (44.7%)
	*No*	15 (13.2%)
	*No formal team exists but nutritional management is aided by regular communication between disciplines*	36 (31.5%)
	*I Don’t know*	12 (10.5%)
**Questions**	**Answers**	**Total** **(*n* = 51)**
(3) Which of the following members included in the nutrition support team in your hospital?**	*Physician*	49 (96.1%)
	*Dietitian*	51 (100.0%)
	*Pharmacist*	43 (84.3%)
	*Nurses*	38 (74.5%)
(4) How frequent does the nutrition support team meet to discuss patient management?	*Daily*	17 (33.3%)
	*Once a week*	13 (25.5%)
	*Once every other week*	3 (5.9%)
	*Once monthly*	5 (9.8%)
	*Only as needed and case by case*	7 (13.7%)
	*I Don’t know*	6 (11.8%)
(5) Does your hospital’s nutrition support team review and report on service performance, quality indicators, patient’s outcome data, and adverse events related to nutrition support therapies?	*Yes*	37 (72.5%)
	*No*	4 (7.8%)
	*I don’t Know*	10 (19.6%)
(6) In your opinion, what are the important barriers in forming a dedicated nutrition support team at your institution?**	*Lack of physicians with interest and qualifications to direct such team*	51 (100.0%)
	*Lack of qualified and dedicated nutrition support pharmacists*	47 (92.2%)
	*Lack of qualified and dedicated nutrition support dietitians*	27 (52.9%)
	*No or little incentives and appreciation of the value of such team by the hospital administration*	49 (96.1%)
	*None*	34 (66.7%)

Data are presented as numbers and percentages. *Only participants who answered Yes (*n* = 51) to this question were allowed to proceed to the following questions related to nutrition support team. **Percentages don’t add to 100 because participants were allowed to choose more than one option.

### Confidence level in practicing nutritional support

When the participants were asked to rate their confidence level in practicing EN, the dietitians had the highest score (8.66 ± 1.63). The confidence level for practicing EN significantly differed (p < 0.01) between the professions (Table 5). Meanwhile, the pharmacists had the highest confidence score (7.36 ± 1.27) in practicing PN; however, no significant difference in the confidence score was observed across the professions (*p* > 0.05). The mean confidence level of all respondents was significantly higher for EN practice (7.7 ± 2.3) than for PN practice (6.1 ± 2.5) (*p* < 0.01).

**TABLE 5:** Comparison of the perception of health care providers related to their confidence level in practicing nutrition support based on their profession, nutritional qualifications, region, years of experience type and capacity of health care facility they work in.

Profession	Physicians (*n* = 38)			Dietitians (*n* = 62)			Pharmacist (*n* = 14)		*p*-value
	**Mean (± SD)**								
(1) Confidence score in practicing EN	7.74 (± 1.82)			8.66 (± 1.63)			3.86 (± 2.24)		0.001*
(2) Confidence score in practicing PN	6.10 (± 2.57)			5.85 (± 2.85)			7.36 (± 1.27)		0.140
Nutritional Qualification*	Yes (*n* = 36)				No (*n* = 78)				*0*-Value
	**Mean (± SD)**								
(1) Confidence score in practicing EN	8.8 (± 1.8)				7.2 (± 2.4)				0.001*
(2) Confidence score in practicing PN	7.5 (± 1.9)				5.4 (± 2.5)				0.001*
Region	Western (*n* = 82)	Eastern (*n* = 10)		Central (*n* = 18)	Southern (*n* = 3)		Northern (*n* = 0)		*p*-Value
	**Mean (± SD)**								
(1) Confidence score in practicing EN	7.7 (± 2.4)	8.2 (± 1.7)		7 (± 2.8)	9.0 (± 0.0)		-		0.638
(2) Confidence score in practicing PN	6.1 (± 2.6)	6.0 (± 2.8)		5.4 (± 2.1)	7 (0.0)		-		0.743
Years of experience	Newly graduate (*n* = 11)		2–5 years (*n* = 43)		6–10 years (*n* = 29)		More than 10 years (*n* = 38)		*p*-Value
	**Mean (± SD)**								
(1) Confidence score in practicing EN	7.7 (± 1.7)		7.1 (± 2.5)		7.9 (± 2.2)		8.2 (± 2.1)		0.155
(2) Confidence score in practicing PN	5.7 (± 3.0)		5.3 (± 2.5)		6.8 (± 2.4)		6.3 (± 2.5)		0.086
Type of health care facility	MOH (*n* = 31)	Military hospitals (*n* = 12)	University teaching hospitals (*n* = 23)	Specialized hospitals (*n* = 15)	National guard hospitals (*n* = 7)	Medical cities (*n* = 6)	Security Forces Hospital (*n* = 2)	Private hospitals (*n* = 18)	*p*-Value
	**Mean (± SD)**								
(1) Confidence score in practicing EN	7.7 (± 1.9)	7.4 (± 2.6)	6.8 (± 2.7)	7.6 (± 2.7)	7.5 (± 2.9)	8.6 (± 1.6)	9 (0.0)	8.9 (± 1.4)	0.202
(2) Confidence score in practicing PN	5.1 (± 3.0)	7.5 (± 2.5)	6.8 (± 1.5)	5.8 (± 2.1)	6.4 (± 2.0)	6.0 (± 1.5)	2 (0.0)	5.8 (± 3.1)	0.128
Capacity of health care facility	<100 beds (*n* = 11)		100–250 beds (*n* = 17)	251–500 beds (*n* = 41)		>500 bed (*n* = 36)		Don’t know (*n* = 9)	*p*-Value
	**Mean (± SD)**								
(1) Confidence score in practicing EN	6.7 (± 2.6)		8.1 (± 1.8)	8.2 (± 2.2)		7.2 (± 2.5)	8.3 (± 1.8)		0.127
(2) Confidence score in practicing PN	6.5 (± 1.6)		5.2 (± 3.6)	5.5 (± 2.2)		6.9 (± 2.2)	6.1(± 3.2)		0.131

Participants were asked to rate their confidence in practicing nutrition support and discussing the patient suitability for it with other clinicians on a scale of 1–10. Kruskal–Wallis test was conducted to compare the mean confidence score between the categories. Mann–Whitney U-test was conducted to compare the mean confidence score between the two categories. *p-value is statistically significant at <0.05 level.

### Factors influencing the confidence level

In the univariate analysis (Table 5), the confidence level for practicing EN and PN significantly differed between the participants with and without nutritional qualification (*p* < 0.01). To evaluate the association between the demographics and confidence level, we conducted a multiple linear regression analysis (Table 6). The regression analysis indicated that the confidence level for practicing EN was significantly influenced by nutritional qualification (β = 0.202, *p* < 0.05), type of healthcare facility (β = 0.210, *p* < 0.05), profession (β = -0.308, *p* < 0.01), and years of experience (β = 0.220, *p* < 0.05). The participants with nutritional qualifications and more years of experience had a higher confidence level for practicing EN than the other participants. Conversely, the confidence level for practicing PN was significantly associated with nutritional qualification (β = 0.398, *p* < 0.01) and region (β = -0.197, *p* < 0.05).

**TABLE 6:** Regression analysis to identify the factors influencing the health care providers confidence when dealing with nutrition support.

Model 1 Outcome variable: Confidence score in practicing enteral nutrition	*R*	*R* ^2^	Adjusted *R*^2^
	0.465	0.216	0.187
***Dependent variable (n* = *114)***	***Beta***	***p-Value***	***Partial correlation***
Nutritional qualification (Yes/No)^a^	0.202	0.027*	0.304
Type of health care facility^a^	0.210	0.022*	0.203
Profession (dietitians, pharmacists, physicians)^a^	−0.308	0.002*	−0.234
Years of experience^a^	0.220	0.023*	0.181
Region^b^	0.77e	0.372	−0.086
**Model 2** **Outcome variable:** **Confidence score in practicing parenteral nutrition**	***R***	***R* ^2^**	**Adjusted *R*^2^**
0.427	0.182	0.167
***Dependent variable (n* = *114)***	***Beta***	***p-Value***	***Partial correlation***
Nutritional qualification (Yes/No)^a^	0.398	0.001*	0.402
Region^a^	−0.197	0.024*	−0.212
Profession (dietitians, pharmacists, physicians)^a^	0.171c	0.057	0.18
Years of experience^b^	0.155c	0.071	0.171
Type of health care facility^b^	0.054c	0.531	0.06

^a^Predictors: (constant).

^b^Excluded variables.

*p-Value is statistically significant at <0.05 level. All models were adjusted for hospital capacity.

## Discussion

The present study aimed to explore various aspects of nutritional support practice in Saudi Arabia. The majority of the participants were dietitians, followed by physicians and pharmacists. Various attitudes in practices across the clinicians were observed. A main finding herein shows that professional qualification and training in nutritional support are essential for promoting practice in hospitals.

### Roles and responsibilities of healthcare professionals in providing nutritional support for hospitalized patients

All participants were relatively involved in determining patient need for nutritional support and selecting the appropriate feeding route. With the application of appropriate nutritional screening, nutritional support clinicians are able to select the correct route of nutritional support without delay. However, the study participants contributed differently to other tasks. Implementing a good nutritional care plan for hospitalized patients greatly depends on the clinician’s ability to determine an adequate caloric and nutritional requirement. The limited involvement of physicians in many tasks found herein could be explained by the lack of knowledge in nutritional support practices. Insufficient nutritional knowledge among Saudi physicians has been previously reported by Alkhaldy (20). Moreover, the pharmacists in this study mainly wrote and monitored PN orders with a minimal role in other tasks. In a previous investigation in Kuwait, pharmacists working at hospitals primarily contributed to technical tasks, such as PN compounding, with limited contribution in direct patient care (21). The role of pharmacists in PN is well recognized. However, pharmacists play different roles in the provision of PN. Their inclusion to NSTs has been reported to reduce metabolic and catheter-related issues compared with PN management by physicians only (22, 23).

Regarding insertion of nutritional support access devices, we found that a large proportion of physicians were involved in inserting EN feeding tubes. Practically, the limited number of personnel privileged to secure nutritional access in hospitals can delay nutritional support initiation as soon as it is needed, which can have detrimental effects especially for patients at a high nutritional risk. Guidelines recommend that enteral feeding tubes or PN vascular accesses should be inserted only by privileged healthcare professionals with appropriate training and qualification (24, 25). Dietitian-led bedside small bowel feeding tube placement and nurse-led PICC line insertion have been described and may improve efficiency and prevent delays in achieving appropriate access especially in hospitals with large volumes of cases and limited resources (24–26). Implementing such concepts requires advanced training, competency, and hospital-specific credentialling to ensure patient safety.

### Awareness and utilization of nutritional support guidelines and protocols

Herein, both ASPEN and ESPEN guidelines were frequently recognized and used in writing nutritional support policies and protocols. Feeding protocols especially in critical illness have been shown to enhance nutritional delivery that meets requirements, resulting in improved outcomes (27). For hospitalized medical inpatients with malnutrition, individualized nutritional support therapy has led to better clinical outcomes and survival compared with standard protocols (28). Among our participants, more dietitians and fewer physicians and pharmacists were involved in writing and updating hospital nutritional support policies and procedures. For such policies to achieve optimal care delivery, involvement of all members of an NST/committee (physicians, nurses, dietitians, and pharmacists) is recommended as a hospital standard by professional societies.

### Nutritional screening and assessment practices for hospitalized patients

All hospitalized patients with poor nutritional intake should have their nutritional risk evaluated before the start of specialized nutritional therapy using a validated screening tool (29). Herein, the awareness and practice of a specific screening tool were low, and most dietitians identified themselves as being involved in screening and assessment. A large cross-sectional survey in the United States found that although hospitals were compliant to malnutrition screening within 24 h of admission, there was variation in the screening tools used (19). Nurses were mostly responsible for screening and dietitians for assessments (19). Although screening is a critical step in the nutritional care algorithm for hospitalized patients, it is the simplest step that can be performed by any trained healthcare professionals. The lack of trained personnel or delay in screening is problematic because it could negatively impact patient outcomes. Additionally, referral for dietetic assessment after screening should be well recognized by most healthcare professionals (i.e., physicians and nurses). The present findings suggest that nurses need to be empowered to refer patients for nutritional assessment.

For nutritional assessment, many respondents used anthropometric measurements, while small proportion, mainly including physicians, reported their use of albumin levels as a nutritional indicator. Regarding the estimation of nutritional requirements, the physicians had minimal involvement in this important step. This could be explained by expectations that clinical dietitians calculate nutritional requirements because of their skillset; however, physicians should play an important role in complementing this step by assessing and communicating patient-related factors that might affect nutritional requirements.

### Nutritional support initiation and monitoring practices

We detected variations in the practitioners’ practices regarding the initiation and monitoring of nutritional support, including the timing and use of the GRV, which can affect optimal provision of nutrition. According to the ASPEN guidelines, early EN must be started within 24–48 h among hemodynamically stable patients in the ICU but who are unable to maintain oral intake (30). However, 39.5% of the respondents waited 3 days. Evidence has shown that early nutritional support in critically ill patients is associated with reduced mortality and infection (30). In terms of PN initiation for well-nourished and stable patients with minimal oral or EN intake, we found that many practitioners waited 3–5 days. Clinical recommendations often advise starting PN (total or supplemental) after 7 days in patients with unmet nutritional requirements (12).

Gastric feeding intolerance is common in critically ill patients. Therefore, monitoring of EN tolerance is essential. Although the GRV is commonly used by many practitioners, it is a poor indicator of EN intolerance (31). The use of multiple parameters, including the GRV and presence of diarrhea, vomiting, and abdominal distention, has been recommended by the ASPEN for evaluating EN intolerance (32).

### Nutritional support plan documentation practices

Data related to nutritional assessment and care plan were the most commonly documented data by the participants. Documentation of nutritional assessment, including nutritional requirements, is a key step and directly affects patient outcomes. Nutritional support process is complex and has many steps that need proper documentation. Improving the documentation quality can help prevent EN/PN-related errors.

### NSTs in Saudi hospitals

The delivery of optimal nutritional support needs a multidisciplinary care team. In this study, the majority of the participants reported that NSTs were very important to the accuracy and efficacy of EN and PN prescription. The existence of NSTs helps improve the quality of care among patients receiving nutritional support. A recent systematic review found that NSTs relatively reduced the rate of catheter-related infections and were significantly correlated with decreased metabolic complications, mortality, and inappropriate utilization of PN (33). Nonetheless, the prevalence of NST implementation in hospitals is decreasing as a result of cutting or saving budgets by healthcare organizations (10). In the United Kingdom, only 60% of hospitals provide nutritional support through a multidisciplinary NST (34). Nearly half of the respondents in the current study had a formal NST comprising physicians, dietitians, pharmacists, and nurses. Typically, NSTs might hold weekly meetings to talk about their operations, specific patients, reported data, and journals (34). The current study reported a great variability in the frequency of NST meetings.

Although NSTs are cost effective (35), their implementation is challenging. The main barriers for implementing NSTs in this study included the lack of physicians with interest, qualified pharmacists, and incentives by hospital administrations. DeLegge et al. reported similar barriers associated with the initiation of NSTs (36). Such barriers must be addressed by future hospitals’ strategies for NST implementation in Saudi Arabia.

### Confidence level among nutritional support practitioners

The confidence level of all study participants was significantly higher for EN practice than for PN practice. It also varied between the healthcare professions. Moreover, the present study identified nutritional qualification and more years of experience as factors enhancing the confidence level in practice. Promoting nutritional support education among healthcare practitioners is important. According to the ASPEN standards of practice, certain minimum qualifications are required for all nutritional support physicians to demonstrate competence to practice in the field of nutrition (37). These qualifications include board certification in a primary specialty, training/experience or certification in nutritional support, participation in institutional nutritional support activities, current clinical responsibility for patients requiring nutritional support therapy, and active membership in a nutritional support professional society. For pharmacists, nutritional support practices vary with the position, education, and practice environment. Certain minimum qualifications are required for all pharmacists involved in nutritional support.

### Strengths and limitations

To our knowledge, this study is the first to describe nutritional support practices in different regions in Saudi Arabia and to identify the factors influencing the confidence of healthcare professionals in practicing EN and PN. In addition, the questionnaire used captured various aspects related to practice. However, a possible limitation of this study is that participation was mostly by dietitians followed by physicians and pharmacists. This could indicate a relatively small number of physicians and pharmacists involved in hospital nutritional support. Therefore, it might be difficult to generalize the results to other healthcare professionals.

## Conclusion

This study explored the current nutritional support practices in Saudi Arabia and identified the factors influencing the confidence of clinicians in practicing EN and PN. It also identified areas where the current hospital standards in Saudi Arabia might be improved and evaluated them against international nutritional support standards. Further, the study provided insights into what Saudi hospitals as a stakeholders can do to improve nutritional support practices. These included using evidence-based hospital-specific protocols, increasing physicians’ awareness about nutritional support guidelines and policies, enhancing nutritional support education and certification among physicians, funding nutritional support committees and national nutritional support training programs, and implementing NSTs in accordance with evidence-based practice guidelines. Clearly, dietitians play a key role in nutritional support; however, the safety and efficacy of care are improved when pharmacists, doctors, and nurses are all involved in the process.

## Data availability statement

The raw data supporting the conclusions of this article will be made available by the authors, without undue reservation.

## Ethics statement

The studies involving human participants were reviewed and approved by Unit of the Biomedical Ethics Research Committee at King Abdulaziz University in Jeddah, Saudi Arabia (HA-02-J-008). The patients/participants provided their written informed consent to participate in this study.

## Author contributions

SA: conceptualization, methodology, formal analysis, and writing–original draft. SZ: conceptualization, methodology, formal analysis, investigation, and writing—review and editing. RM: methodology, investigation, and writing—review and editing. HJ: conceptualization, investigation, and writing—review and editing. All authors critically revised the manuscript and approved the final version of the manuscript.
