# Supplementary material for: Exploring the practice of nutritional support during hospitalization across physicians, dietitians, and pharmacists based in Saudi Arabia
Source: Front Nutr. 2023 May 24;10:1149727. doi: 10.3389/fnut.2023.1149727 (PMC10244628; doi:10.3389/fnut.2023.1149727)
Supplement: Supplementary file 1 [file Data_Sheet_1.docx]

**Supplementary Tables**

**Table S1:** Nutrition support-related activities performed by healthcare providers

| **Which of the following nutrition support-related activities you are involved in?*** | **Physicians (n=38)** | **Dietitians (n=62)** | **Pharmacists**  **(n=14)** |
| --- | --- | --- | --- |
|  | **n (%)** | | |
| 1. ***Determining the patient’s need for specialized nutrition support and appropriate route (EN and/or PN)*** | **24 (63.1%)** | **53**  **(85.5%)** | **8**  **(57.1%)** |
| 1. ***Calculating nutritional requirements*** | **15**  **(39.5%)** | **62**  **(100%)** | **12**  **(85.7%)** |
| 1. ***Calculating fluid and electrolyte requirements*** | **14**  **(36.8%)** | **52**  **(83.8%)** | **12**  **(85.7%)** |
| 1. ***Order writing for enteral nutrition*** | **23**  **(60.5%)** | **50**  **(80.6%)** | **2**  **(14.2%)** |
| 1. ***Order writing for parenteral nutrition*** | **14**  **(36.8%)** | **15**  **(24.1%)** | **12**  **(85.7%)** |
| 1. ***Selection of oral nutrition supplement/formula*** | **17**  **(44.7%)** | **58**  **(93.5%)** | **1**  **(7.1%)** |
| 1. ***Insertion of nasogastric tube for EN administration*** | **22**  **(65.7%)** | **8**  **(12.9%)** | **0**  **(0%)** |
| 1. ***Insertion of PEG/PEJ tubes for EN administration*** | **23**  **(65.7%)** | **4**  **(6.4%)** | **0**  **(0%)** |
| 1. ***Placing vascular access devices for PN administration*** | **5**  **(13.1%)** | **3**  **(4.8%)** | **0**  **(0%)** |
| 1. ***Initiating order of nutrition support*** | **27**  **(71.1%)** | **45**  **(72.5%)** | **7**  **(50%)** |
| 1. ***Monitoring enteral feeding intake and tolerance*** | **15**  **(39.5%)** | **57**  **(91.9%)** | **0**  **(0%)** |
| 1. ***Monitoring parenteral nutrition intake and tolerance*** | **12 (31.5%)** | **31**  **(50%)** | **14**  **(100%)** |
| 1. ***Educating patient regarding nutrition support plan*** | **15**  **(39.4)** | **62 (100%)** | **4**  **(28.5)** |

Data are presented as numbers and percentages

The above table shows the frequencies of the nutrition support tasks performed by each profession

*Participants were allowed to choose more than one option.

**Table S2:** Nutrition screening practices in Saudi hospitals as reported by healthcare providers

| **Questions** | **Answers** | **Total**  **(n=114)** |
| --- | --- | --- |
| 1. **Which of the following nutrition screening tools is routinely being used in your hospital*** | *Malnutrition Universal Screening Tool (MUST)* | **26**  **(22.8%)** |
|  | *Nutrition Risk Screening (NRS 2002)* | **32**  **(28.07%)** |
|  | *Mini Nutritional Assessment (MNA)* | **6**  **(5.26%)** |
|  | *Short Nutritional Assessment Questionnaire (SNAQ)* | **5**  **(4.39%)** |
|  | *Malnutrition Screening Tool (MST)* | **4**  **(3.51%)** |
|  | *Subjective Global Assessment (SGA)* | **12**  **(10.53%)** |
|  | *Centers for Disease Control and Prevention (CDC) growth charts* | **14**  **(12.28%)** |
|  | *World Health Organization growth charts* | **19**  **(16.67%)** |
|  | *Z score* | **12**  **(10.53%)** |
|  | *I Don’t know* | **41**  **(35.96%)** |
|  | *Other* | **11**  **(9.65%)** |
| 1. **Is screening for malnutrition routinely done at your hospital ward**** | *Yes* | **55**  **(48.2%)** |
|  | *No* | **32**  **(28.1%)** |
|  | *I Don’t know* | **27**  **(23.7%)** |
| **Questions** | **Answers** | **Total**  **(n=55)** |
| 1. **Who is primarily responsible for the initial screening for malnutrition in your practice setting?** | *Dietitian* | **25**  **(45.4%)** |
|  | *Physician* | **9**  **(16.4%)** |
|  | *Nurse* | **21**  **(38.2%)** |
| 1. **When is nutrition screening routinely conducted in your practice setting?** | *On admission only* | **13**  **(23.6%)** |
|  | *On admission then re-screened periodically* | **42**  **(76.4%)** |

Data are presented as numbers and percentage

*Percentages don’t add to 100 because participants were allowed to choose more than one option.

**Only participants who answered Yes (n=55) to this question were allowed to proceed to the following questions, participants who answered No or I don’t know were exempted from questions 4 to 6

**Table S3:** Nutrition assessment practices in Saudi hospitals as reported by healthcare providers

| **Questions** | **Answers** | **Physicians (n=38)** | **Dietitians (n=62)** | **Pharmacists**  **(n=14)** | **Total**  **(n=114)** |
| --- | --- | --- | --- | --- | --- |
|  |  | **n (%)** | | | |
| 1. **At your institution, what is the process of referral for dietitian assessment and intervention in patients who are at nutritional risk?** | *Dietitians assess all newly admitted patients regardless of their nutritional risk* | **9**  **(23.7%)** | **22**  **(35.5%)** | **4**  **(28.6%)** | **35**  **(30.7%)** |
|  | *Dietitians’ referrals are done by nurses after they conducted initial screening* | **6**  **(15.8%)** | **14**  **(22.6%)** | **0**  **(0%)** | **20**  **(17.5%)** |
|  | *Dietitians’ referrals need to be ordered by a physician* | **19**  **(50%)** | **24**  **(38.7%)** | **5**  **(35.7%)** | **48**  **(42.1%)** |
|  | *Other* | **3**  **(7.9%)** | **0**  **(0%)** | **5**  **(35.7%)** | **8**  **(7.0%)** |
|  | *I Don’t know* | **1**  **(2.7%)** | **2**  **(3.2%)** | **0**  **(0%)** | **3**  **(2.6%)** |
| 1. **Which of the following do you mostly rely on as a clinical indicator for nutritional status?** | *Albumin* | **24**  **(63.1%)** | **4**  **(6.5%)** | **5**  **(35.7%)** | **33**  **(28.9%)** |
|  | *Prealbumin* | **4**  **(10.5%)** | **4**  **(6.5%)** | **1**  **(7.1%)** | **9**  **(7.9%)** |
|  | *Transferrin* | **0**  **(0.0%)** | **0**  **(0.0%)** | **0**  **(0.0%)** | **0**  **(0.0%)** |
|  | *Anthropometry* | **8**  **(21.0%)** | **51**  **(82.2%)** | **8**  **(57.1%)** | **67**  **(58.8%)** |
|  | *Other* | **2**  **(5.2%)** | **3**  **(4.8%)** | **0**  **(0.0%)** | **5**  **(4.4%)** |
|  | *I Don’t know* | **0**  **(0.0%)** | **0**  **(0.0%)** | **0**  **(0.0%)** | **0**  **(0.0%)** |
| 1. **Which of the following does apply to your current practice regarding estimation of patient caloric requirements?** | *I am not involved in calculating caloric requirements (done by other team members)* | **16**  **(42.1%)** | **1**  **(1.61)** | **2**  **(14.2%)** | **19**  **(16.7%)** |
|  | *I mostly use simple weight-based equations* | **17**  **(44.7%)** | **47**  **(75.8)** | **7**  **(50%)** | **71**  **(62.3%)** |
|  | *I mostly use predictive equations such as Harris Benedict* | **2**  **(5.2%)** | **14 (22.5%)** | **5**  **(35.7%)** | **21**  **(18.4%)** |
|  | *I have access to indirect calorimetry when needed* | **3**  **(7.8%)** | **0**  **(0.0%)** | **0**  **(0.0%)** | **3**  **(2.6%)** |

Data are presented as number and percentages

**Table S4:**

|  | **Confidence score in practicing EN** | **Confidence score in practicing PN** | **P-value** |
| --- | --- | --- | --- |
|  | **Mean (**±**SD)** | |  |
| **Physicians** | 7.74 (±1.82) | 6.10 (±2.57) | **0.001*** |
| **Dietitians** | 8.66 (±1.63) | 5.85 (±2.85) | **0.001*** |
| **Pharmacists** | 3.86 (±2.24) | 7.36 (±1.27) | **0.00*** |
| **All** | 7.7 (±2.3) | 6.1 (±2.5) | **0.001*** |

Mann-Whitney U-test was conducted to compare the mean confidence score

*P value is statistically significant at < 0.05 level.
