# Supplementary material for: Exploring the practice of nutritional support during hospitalization across physicians, dietitians, and pharmacists based in Saudi Arabia
Source: Front Nutr. 2023 May 24;10:1149727. doi: 10.3389/fnut.2023.1149727 (PMC10244628; doi:10.3389/fnut.2023.1149727)
Supplement: Supplementary file 2 [file Data_Sheet_2.pdf]

Assessing The Current Practice of Nutrition Support in Hospitalized Patients in Saudi Arabia

You are being invited to take part in this research study conducted by researchers from King Abdulaziz University and Taibah University. Before you decide to participate, it is important for you to understand why the research is being done. Please take your time to read the following information carefully and decide whether or not you wish to participate.

Nutrition support is considered an integral part in the care for hospitalized patients, particularly those with critical illness. Malnutrition is being reported in approximately 20% to 50% of hospitalized patients. Appropriate practices by healthcare professionals in identifying and managing malnutrition in hospitals is essential. Therefore, evidence-based nutrition support practices will ensure the quality and safety of nutrition support delivery. In Saudi Arabia, nutrition support practices by healthcare professionals are not yet described. The aim of this study is to investigate the current practice of nutrition support among physicians, dietitians, and pharmacists currently working in Saudi hospitals.

If you decided to participate in this study, please answer the questionnaire by selecting the option that best corresponds to your current practice.

All the information that will be collected from you in this survey will be kept anonymous and strictly confidential. Your participation in this study is entirely voluntary, and you may refuse to participate or withdraw from the study at any time.

Thank you for your participation.

Contacts for further information: smajabnoor@kau.edu.sa

*Required

1. Have you filled this Questionnaire before? *

Mark only one oval.

- Yes
- No

2. Do you agree on participation in this study by filling this questionnaire? *

Mark only one oval.

- Yes
- No

3. Do you have some involvement in nutrition support for hospitalized patients? (Nutrition support is a part of the medical therapy which help in treating and preventing malnutrition. It include enteral and parenteral nutrition) *

Mark only one oval.

- Yes
- No

Demographics

4. Specify what region are you based in? *

Mark only one oval.

- Western region
- Eastern region
- Central region
- Southern region
- Northern region

5. Specify your type of practice? *

Mark only one oval.

- Dietitian
- Pharmacist
- Gastroenterologist (adults)
- Gastroenterologist (paediatrics)
- General surgeon or subspecialty
- Intensivist (adults)
- Intensivist (paediatrics)
- Intensivist (neonatal)
- Internal medicine or subspecialty
- Paediatrics or subspecialty
- Other: ____________________

6. If you are a dietitian or pharmacist, which hospital ward do you typically cover? *If you are a physician please skip this question. (Choose all that apply)

Tick all that apply.

- [ ] Medical ward (adults)
- [ ] Surgical ward (adults)
- [ ] Paediatric ward
- [ ] Obstetrics / Gynaecology ward
- [ ] Medical intensive care
- [ ] Surgical intensive care
- [ ] Paediatric intensive care
- [ ] Neonatal intensive care

7. Specify your clinical practice setting? *

Mark only one oval.

- Ministry of Health (MOH) hospitals
- Military hospitals
- University teaching hospitals
- Specialized hospitals (King Faisal Specialist Hospital and Research Centre)
- National guard hospitals
- Medical cities (e.g. Prince Sultan Medical City)
- Private hospitals
- Other: ______________________

8. For how long have you practiced? *

Mark only one oval.

- Newly graduated
- 2 to 5 years
- 6 to 10 years
- More than 10 years

9. Approximately how many beds are there in your current hospital/institution? *

Mark only one oval.

- <100 beds
- 100-250 beds
- 251-500 beds
- >500 bed
- Don't know

10. Which of the following nutrition support related activities you are involved in? (Choose all that apply) *

Tick all that apply.

- [ ] Determining the patient's need for specialized nutrition support and appropriate route (EN and/or PN)
- [ ] Calculating nutritional requirements
- [ ] Calculating fluid and electrolyte requirements
- [ ] Order writing for enteral nutrition
- [ ] Order writing for parenteral nutrition
- [ ] Selection of oral nutrition supplement/formula
- [ ] Insertion of nasogastric tube for EN administration
- [ ] Insertion of PEG/PEJ tubes for EN administration
- [ ] Placing vascular access devices for PN administration
- [ ] Initiating order of nutrition support
- [ ] Monitoring enteral feeding intake and tolerance
- [ ] Monitoring parenteral nutrition intake and tolerance
- [ ] Educating patient regarding nutrition support plan

11. Have you received a nutrition support specific additional training or qualification during your postgraduate studies, residency program, or fellowship? (Choose all that apply) *

Tick all that apply.

- [ ] Master of clinical nutrition with focus on nutrition support
- [ ] Fellowship in clinical nutrition
- [ ] ASPEN nutrition support certification
- [ ] ESPEN diploma in clinical nutrition and metabolism
- [ ] None
- [ ] Other: ____________________

Nutrition support team - 1

12. Does your hospital have an established multidisciplinary nutrition support team that is currently active? (Nutrition support team is a multidisciplinary team that mainly consists of physicians, registered dietitians, clinical pharmacists, and nurses. The main role of this team is to provide consultation for the management of enteral and parenteral nutrition). *

Mark only one oval.

- ( ) Yes
- ( ) No *Skip to question 16*
- ( ) No formal team exists but nutritional management is aided by regular communication between disciplines (interprofessional) *Skip to question 16*
- ( ) Don't know *Skip to question 16*

Nutrition support team - 2

13. Which of the following members included in the nutrition support team in your hospital? (Choose all that apply) *

Tick all that apply.

- [ ] Physician
- [ ] Dietitian
- [ ] Pharmacist
- [ ] Nurse
- [ ] Other: ____________________

14. How frequent does the nutrition support team meet to discuss patient management? *

Mark only one oval.

- ( ) Daily
- ( ) Once a week
- ( ) Once every other week
- ( ) Once monthly
- ( ) Only as needed and case by case
- ( ) Don't know

15. Does your hospital's nutrition support team review and report on service performance, quality indicators, patient's outcome data, and adverse events related to nutrition support therapies? *

Mark only one oval.

- Yes
- No
- Don't know

Nutrition support team - 3

16. In your opinion, what are the important barriers in forming a dedicated nutrition support team at your institution? (Choose all that apply) *

Tick all that apply.

- [ ] Lack of physicians with interest and qualifications to direct such team
- [ ] Lack of qualified and dedicated nutrition support pharmacists
- [ ] Lack of qualified and dedicated nutrition support dietitians
- [ ] No or little incentives and appreciation of the value of such team by the hospital administration
- [ ] None

17. In your opinion, how important is having nutrition support team to the accuracy and efficacy of nutritional prescription for hospitalized patients? *

Mark only one oval.

- Very important
- Somewhat important
- Not important

Perceptions related to the use of established nutrition support guidelines - 1

18. Are you familiar with the American Society for Parenteral and Enteral Nutrition (ASPEN) and/or the European Society of Clinical Nutrition and Metabolism (ESPEN) guidelines for nutrition support? *

Mark only one oval.

- Yes
- No

19. Does your hospital have written policies and procedures for the provision of nutrition support therapy (i.e. timing and route of feed initiation, formula selection, assessing patient's nutrient requirements, and assessing feeding intolerance)? *

Mark only one oval.

- Yes
- No *Skip to question 22*
- Don't know *Skip to question 22*

Perceptions related to the use of established nutrition support guidelines - 2

20. Are you involved in writing and updating the hospital's nutrition support policies and procedures? *

Mark only one oval.

- Yes
- No

21. Which of the following guidelines for nutrition support are you using as a reference in your institution? (Choose all that apply) *

Tick all that apply.

- [ ] ASPEN
- [ ] ESPEN
- [ ] Don't know
- [ ] Other: ______________________

Nutrition screening and assessment - 1

22. Is screening for malnutrition routinely done at your hospital ward? *

Mark only one oval.

- ( ) Yes
- ( ) No *Skip to question 26*
- ( ) Don't know *Skip to question 26*

Nutrition screening and assessment - 2

23. Who is primarily responsible for the initial screening for malnutrition in your practice setting? *

Mark only one oval.

- ( ) Dietitian
- ( ) Physician
- ( ) Nurse
- ( ) Don't know
- ( ) Other: ______________________

24. When is nutrition screening routinely conducted in your practice setting? *

Mark only one oval.

- On admission only
- On admission then re-screened periodically
- Don't know
- Other: ____________________

25. Is a validated nutrition screening tool for detecting malnutrition being used among hospitalized patients in your hospital? *

Mark only one oval.

- Yes
- No
- Don't know

Nutrition screening and assessment - 3

26. Which of the following nutrition screening tools is routinely being used in your hospital? (Choose all that apply) *

Tick all that apply.

- [ ] Malnutrition Universal Screening Tool (MUST)
- [ ] Nutrition Risk Screening (NRS 2002)
- [ ] Mini Nutritional Assessment (MNA)
- [ ] Short Nutritional Assessment Questionnaire (SNAQ)
- [ ] Malnutrition Screening Tool (MST)
- [ ] Subjective Global Assessment (SGA)
- [ ] Centers for Disease Control and Prevention (CDC) growth charts
- [ ] World Health Organization growth charts
- [ ] Z score
- [ ] Don't know
- [ ] Other: ____________________

27. At your institution, what is the process of referral for dietitian assessment and intervention in patients who are at nutritional risk? *

Mark only one oval.

- Dietitians assess all newly admitted patients regardless of their nutritional risk
- Dietitians referrals are done by nurses after they conducted initial screening
- Dietitians referrals need to be ordered by a physician
- Don't know
- Other: ____________________

28. Which of the following do you mostly rely on as a clinical indicator for nutritional status? *

Mark only one oval.

- Albumin
- Pre-albumin
- Transferrin
- Anthropometry
- Other: ____________________

29. Which of the following does apply to your current practice regarding estimation of patients' caloric requirements? *

Mark only one oval.

- I am not involved in calculating caloric requirements (done by other team members)
- I mostly use simple weight-based equations
- I mostly use predictive equations such as Harris Benedict
- I have access to indirect calorimetry when needed

Practices related to the initiation and monitoring of nutrition support

30. In your practice, in a critically-ill and hemodynamically stable patient, after how many days of nil per oral (NPO) status, would you wait before the use of artificial nutrition support? *

Mark only one oval.

- 1 day
- 2 days
- 3 days
- Not applicable in my practice
- Other: ______________________

31. In your practice, after how many days of minimal oral intake or enteral nutrition (less than 50% of estimated caloric requirements) by a well-nourished, stable patient, would you initiate parenteral nutrition? *

Mark only one oval.

- 3-5 days
- 7 days
- 14 days
- Don’t know
- Not applicable in my practice

32. In your practice, what would you do if a patient experiences a few nausea or vomiting with gastric tube feeding? (Choose all that apply) *

Tick all that apply.

- [ ] Stop tube feeding
- [ ] Slow tube feeding
- [ ] Give promotility agent
- [ ] Check gastric residual volume
- [ ] Check tube placement
- [ ] Place tube to suction
- [ ] Advance tube
- [ ] Switch to parenteral feeding
- [ ] Consult a specialist
- [ ] Perform a physical examination
- [ ] Elevate the head of the bed
- [ ] Check gastric emptying study
- [ ] Give IV fluids
- [ ] Don't know
- [ ] Other: ____________________

33. In your practice, do you routinely measure gastric residual volume (GRV) in patients receiving enteral nutrition as a measure of enteral feeding intolerance? *

Mark only one oval.

- ( ) Yes
- ( ) No
- ( ) Don't know

Hospital documentation of nutritional support data

34. At your institution, which of the following nutritional data is documented or carried out using the hospital's electronic health record system? (Choose all that apply) *

Tick all that apply.

- [ ] Nutrition screening data
- [ ] Nutrition assessment data
- [ ] Nutrition care plan
- [ ] Enteral nutrition order entry
- [ ] Parenteral nutrition order entry
- [ ] Nutrition monitoring and evaluation data
- [ ] None of the above (all are done manually)

Perceptions related to the confidence level in practicing nutrition support

35. On a scale of 1 to 10, how do you rate your confidence in practicing enteral nutrition and discussing the patient suitability for it with other clinicians? *

Mark only one oval.

	Lowest
1	◯
2	◯
3	◯
4	◯
5	◯
6	◯
7	◯
8	◯
9	◯
10	◯
	Highest

36. On a scale of 1 to 10, how do you rate your confidence in practicing parenteral nutrition and discussing the patient suitability for it with other clinicians? *

Mark only one oval.

Lowest

1 ◯

2 ◯

3 ◯

4 ◯

5 ◯

6 ◯

7 ◯

8 ◯

9 ◯

10 ◯

Highest

This content is neither created nor endorsed by Google.

Google Forms
